# Video Consultation as an Adequate Alternative to Face-to-Face Consultation in Continuous Positive Airway Pressure Use for Newly Diagnosed Patients With Obstructive Sleep Apnea: Randomized Controlled Trial

**DOI:** 10.2196/20779

**Published:** 2021-05-11

**Authors:** Laura Kooij, Petra JE Vos, Antoon Dijkstra, Elisabeth A Roovers, Wim H van Harten

**Affiliations:** 1 Rijnstate Arnhem Netherlands; 2 Department of Health Technology and Services Research University of Twente Enschede Netherlands; 3 Pulmonary Department Rijnstate Arnhem Netherlands; 4 Division of Psychosocial Research and Epidemiology Netherlands Cancer Institute Amsterdam Netherlands

**Keywords:** video consultation, eHealth, obstructive sleep apnea, continuous positive airway pressure, randomized controlled trial

## Abstract

**Background:**

The effectiveness of continuous positive airway pressure (CPAP) is dependent on the degree of use, so adherence is essential. Cognitive components (eg, self-efficacy) and support during treatment have been found to be important in CPAP use. Video consultation may be useful to support patients during treatment. So far, video consultation has rarely been evaluated in thorough controlled research, with only a limited number of outcomes assessed.

**Objective:**

The aim of the study was to evaluate the superiority of video consultation over face-to-face consultation for patients with obstructive sleep apnea (OSA) on CPAP use (minutes per night), adherence, self-efficacy, risk outcomes, outcome expectancies, expectations and experiences with video consultation, and satisfaction of patients and nurses.

**Methods:**

A randomized controlled trial was conducted with an intervention (video consultation) and a usual care group (face-to-face consultation). Patients with confirmed OSA (apnea-hypopnea index >15), requiring CPAP treatment, no history of CPAP treatment, having access to a tablet or smartphone, and proficient in the Dutch language were recruited from a large teaching hospital. CPAP use was monitored remotely, with short-term (weeks 1 to 4) and long-term (week 4, week 12, and week 24) assessments. Questionnaires were completed at baseline and after 4 weeks on self-efficacy, risk perception, outcome expectancies (Self-Efficacy Measure for Sleep Apnea), expectations and experiences with video consultation (covering constructs of the unified theory of acceptance and use of technology), and satisfaction. Nurse satisfaction was evaluated using questionnaires.

**Results:**

A total of 140 patients were randomized (1:1 allocation). The use of video consultation for OSA patients does not lead to superior results on CPAP use and adherence compared with face-to-face consultation. A significant difference in change over time was found between groups for short-term (*P*-interaction=.008) but not long-term (*P*-interaction=.68) CPAP use. CPAP use decreased in the long term (*P*=.008), but no significant difference was found between groups (*P*=.09). Change over time for adherence was not significantly different in the short term (*P*-interaction=.17) or long term (*P*-interaction=.51). A relation was found between CPAP use and self-efficacy (*P*=.001), regardless of the intervention arm (*P*=.25). No significant difference between groups was found for outcome expectancies (*P*=.64), self-efficacy (*P*=.41), and risk perception (*P*=.30). The experiences were positive, and 95% (60/63) intended to keep using video consultation. Patients in both groups rated the consultations on average with an 8.4. Overall, nurses (n=3) were satisfied with the video consultation system.

**Conclusions:**

Support of OSA patients with video consultation does not lead to superior results on CPAP use and adherence compared with face-to-face consultation. The findings of this research suggest that self-efficacy is an important factor in improving CPAP use and that video consultation may be a feasible way to support patients starting CPAP. Future research should focus on blended care approaches in which self-efficacy receives greater emphasis.

**Trial Registration:**

Clinicaltrials.gov NCT04563169; https://clinicaltrials.gov/show/NCT04563169

## Introduction

Telemedicine is increasingly used to support self-management in chronic diseases and is defined as the use of information and communication technology to deliver health care at a distance [1], but so far we see little evidence in this field. Nevertheless, telemedicine solutions are used for patients with obstructive sleep apnea (OSA) for example, for monitoring, education, and consultation [2]. OSA is considered a chronic disease [1,3]; it is a sleep disorder that affects at least 2% to 4% of the adult population [4] and is characterized by repeated episodes of full or partial occlusion of the upper airway during sleep [4,5]. This condition can have multiple effects on patients’ health such as cognitive dysfunction [4], decrease in health-related quality of life [4,6], increase in cardiovascular disease risk, and sleepiness during the daytime [6]. The severity is often determined with the apnea-hypopnea index (AHI) [4], which represents the number of apneas and hypopneas per hour [4] and is classified as mild (5 to 15 per hour), moderate (15 to 30 per hour) or severe (>30 per hour) [7]. Continuous positive airway pressure (CPAP) is the preferred treatment [6], especially for moderate to severe OSA [5]. CPAP prevents the airway from narrowing or collapsing by applying a positive pressure via a nasal mask during sleep [8] and is tailored to each patient [9]. As the effectiveness of CPAP is dependent on use [5,10], treatment adherence is essential. Cognitive components, mainly based on the social cognitive theory [11], are becoming increasingly important in predicting CPAP use [12-14]. Support during treatment [15], tailored interventions [16], and closer follow-up [17] can also positively affect adherence.

Video consultation may be a useful way to support patients [1,17,18] during treatment and is defined as a “technology used to realize a real-time visual and audio patient assessment at a distance” [19]. Video consultation has been beneficial in chronic conditions (eg, diabetes [20,21] and cancer [19,22]) and in care for OSA patients [17,18]. The use for OSA patients may be promising, especially since physical examination is not always needed [1], and CPAP use can already be monitored remotely [23]. However, the evidence on the effectiveness for OSA patients is still limited [24]. Previous studies were narrowly focused, with mainly adherence [18,25] and satisfaction [17,18,26] being assessed. Although cognitive components, (eg, self-efficacy and outcome expectancies) are found to be important elements for CPAP use [13,14,27], there is a lack of evidence about these effects on video consultation for OSA patients. Previous research on OSA patients also mainly evaluated the use of video consultation for initial contact with health care professionals focused on diagnosis, treatment plans [18,26], or for training purposes [17]. The use of video consultation may be particularly relevant during follow-up (after an initial face-to-face contact) for newly diagnosed patients, since support during treatment is important [15] and successful CPAP use is often determined at an early stage of treatment [28].

Only a limited number of randomized controlled trials (RCTs) were conducted [17,25,26,29], with only one fully powered trial [29]. In a study by Smith et al [25], video consultation was used by nurses for patients who were nonadherent during the first 3 months of treatment. One group of patients received specific information (n=10) about CPAP and one group (n=9) generic information. Both adherence and satisfaction were higher in the intervention group (*P*=.003). Isetta et al [29] conducted a multicenter RCT with patients receiving access to either a telemedicine program (n=69) with video consultations or usual care (hospital visits, n=70). Although the telemedicine approach was assumed to be more cost-effective, CPAP adherence was equivalent after 6 months [29]. Video consultation was also used for initial contact before starting treatment, with mixed results. The use of video consultation for training purposes did not lead to a difference in knowledge [17]. Also, no significant differences in satisfaction and CPAP adherence were found after 14 days for new OSA patients starting CPAP treatment [18]. Adherence rates were found to be higher after 6 months for patients who received their initial consultation face-to-face than via video consultation. However, statistically significant difference was not reported [26].

Video consultation is often found to be as effective as face-to-face consultation in terms of CPAP use [18,29]. Previous studies often focused on newly diagnosed patients before the start of treatment [17,18,26], with generally small sample sizes [17,25,26]. Patients are satisfied with video consultation [17,18,25], and it may be a promising way to deliver more convenient care with indirect benefits for patients (eg, less travel time) [24]. Additionally, remote monitoring [30] and patient support treatment [31] can positively affect CPAP use [30,31]. Therefore, it may be expected that video consultation in combination with remotely monitoring CPAP use, consultation with nurses, and the indirect benefits of video consultation (eg, less travel time) [24] may improve CPAP use. Cognitive components (eg, self-efficacy) are also found to be important elements for CPAP use [13,14,27], but evaluation in combination with video consultation is lacking [24]. More evidence about the technology being used and health care professionals’ perceptions is also needed to ensure successful implementations [17]. Such knowledge is essential because the use of video consultation is increasing, but evidence is still lacking and powered studies are needed [24].

Therefore, the objective of this paper is to evaluate the superiority of video consultation versus face-to-face consultation for patients with OSA on CPAP use (minutes per night), CPAP adherence, self-efficacy, risk perception, outcome expectancy, video consultation expectations and experiences with technology, and the satisfaction of patients and nurses.

## Methods

### Study Design

We conducted a nonblinded RCT with an intervention group (video consultation) and a usual care group (face-to-face consultation), with 1:1 allocation.

### Recruitment and Participants

Patients were recruited from a large teaching hospital (Rijnstate, Arnhem). To be eligible to participate, patients had to be older than 18 years, be diagnosed with moderate or severe OSA (AHI >15), require CPAP treatment, have no history of CPAP treatment, have access to a tablet or smartphone, and be proficient in the Dutch language. Exclusion criteria were having a psychiatric or cognitive disorder.

### Study Process

Prior to the study, a letter was sent to patients to confirm their appointments (eg, sleep study and consultation with the pulmonologist) including information about the study. During the first face-to-face consultation with the pulmonologist, patients received their treatment plan and information about the study (including information letter and informed consent form). This was followed by instruction about their CPAP treatment. After this consultation, the researcher provided patients with additional information about the study, and they were asked to sign the informed consent form. For reasons of clinical necessity, patients started treatment the same day.

### Randomization

After patients signed informed consent and completed the baseline questionnaire, they were randomized by the researcher to the intervention or usual care group using the software program Research Manager (Cloud9 Software) with block size of 10. The researcher informed the patients about their allocation, and the intervention group received additional information about the video consultation app (Facetalk, Qconferencing) [32]. All participants received a copy of the informed consent form, and a follow-up appointment was planned directly.

### Intervention

The video consultation app Facetalk [32] could be downloaded (for free) from Google Play [33] or the App Store [34]. The first video consultation with a nurse was planned for 1 week after the start of CPAP. Patients received an email with the date, time, and a link to start the video consultation in the app. Three focus points were discussed during the consultations: (1) adherence (>6 hours per night), (2) rest AHI <5 (or <10 if age over 70 years), and (3) (improvements in) symptoms. If these objectives were achieved after 1 week, a new consultation was planned for 3 weeks later (4 weeks after the start). If these objectives were not achieved, video consultations were planned for weekly (until 4 weeks after starting CPAP treatment). After 4 weeks, patients received a questionnaire. See Multimedia Appendix 1 for the study process.

### Usual Care

The usual care group followed the same care process but with face-to-face consultation instead of video consultation. Patients received a confirmation letter with the day and time of their next consultation.

### Outcome Measures

#### Primary Outcome

The primary outcome was CPAP use (minutes per night), monitored remotely with Encore Anywhere (Philips). Conforming to the initial protocol, CPAP use was assessed during the first 4 weeks (short-term). Additionally, we assessed CPAP use after week 4, week 12, and week 24 (long-term).

#### Secondary Outcomes

##### CPAP Adherence

CPAP adherence was defined as CPAP use for at least 5 nights per week for at least 4 hours per night [15,35] and was assessed during the first 4 weeks (short-term) and week 4, week 12, and week 24 (long-term).

##### Treatment Self-Efficacy, Risk Perception, and Outcome Expectancies

The Self-Efficacy Measure for Sleep Apnea (SEMSA) [13] was used to measure cognitive components: self-efficacy, risk perception, and outcome expectancies. The SEMSA is a 26-item scale [13] with subscales: self-efficacy and outcome expectancies each have 9 questions rated on a 4-point scale from not at all true to very true and risk perception has 8 questions rated on a 4-point scale from very low to very high. The mean of the nonmissing item responses was calculated for risk perception, outcome expectancies, and self-efficacy. For the purpose of this study, the SEMSA was translated back (from English into Dutch) and forth (from Dutch into English) by Taalcentrum-VU [36]. In this study, the statements from the published paper were used [13].

##### Relation Between Self-Efficacy, Risk Perception, Outcome Expectancies, and CPAP Use

The relations between CPAP use and self-efficacy, risk perception, and outcome expectancies were assessed. Also, the differences between the intervention and usual care group were analyzed.

##### Expectations and Experiences With Video Consultation

Questions covering constructs of the unified theory of acceptance and use of technology (UTAUT) model [37] were used to measure expectations and experiences with the use of the video consultation system. The UTAUT consists of 4 constructs that influence behavioral intention and behavior—performance expectancy, effort expectancy, social influence, and facilitating conditions [37]. A total of 9 questions were rated on a 7-point scale (1=totally disagree to 7=totally agree).

##### Satisfaction

Patient satisfaction was evaluated with questions about the consultations and information received. Additionally, the intervention group answered questions about the video consultation system. All questions were rated on a 5-point scale (from 1=totally disagree to 5=totally agree). Nurses’ experiences were evaluated using a questionnaire with questions about the video consultation system, satisfaction, and organizational benefits (eg, time and efficiency).

##### Other Parameters

Patient age, marital status, education, experience with internet and internet use, tablet or smartphone skills, and support (with tablet or smartphone use) were assessed via a questionnaire at baseline. Data about comorbidities, AHI, number of consultations, symptoms, and results of the Epworth Sleepiness Scale [38] were obtained from the electronic medical record. This scale is a self-administered questionnaire to examine the perception of daytime sleepiness that has 8 questions about how likely it is to doze off in different situations ranging from 0 to 3. A total score for this scale is calculated by taking the sum of the 8 items. A total of 11 to 12 is considered mild, 13 to 15 moderate, and 16 to 24 severe excessive daytime sleepiness [39]. In this study, a total score of >10 is considered excessive daytime sleepiness.

#### Sample Size Calculation

Since there is no determined clinically relevant difference for CPAP use [40], we assumed that a difference of 1 (SD 2.0) hour per day of average CPAP use (primary outcome) is clinically significant [13,29]. Using a *t* test, alpha of .05, and 80% power, 63 subjects per group (a total of 126) were needed. Correcting for 10% dropout, 70 patients were recruited for each group.

### Statistical Analysis

Data analysis was performed using SPSS (version 22.0, IBM Corp). Descriptive statistics were used to report the baseline characteristics, experiences, expectations, and satisfaction. Linear mixed models were used to analyze differences in CPAP use over time for the intervention and usual care group (interaction term: time × group). All available CPAP use data were used in the analysis, according to the intention-to-treat principle. Differences in adherence over time between groups was analyzed using generalized estimating equations. The relation between CPAP use and risk perception, outcome expectancies, and self-efficacy was analyzed with a linear regression. Normally distributed variables were reported as mean and standard deviation, and statistical differences were tested using an independent samples *t* test. Nonnormally distributed data were reported with medians and interquartile range (25th to 75th percentiles), and differences between groups were analyzed with Mann-Whitney *U* tests.

### Approval and Ethical Considerations

All participants signed a written informed consent form prior to inclusion in the study. The study was approved by the regional medical research ethics committee Commissie Mensgebonden Onderzoek Arnhem–Nijmegen and registered at Clinicaltrials.gov [NCT04563169].

## Results

### Recruitment and Participants

Patients were included from January 2, 2019, until June 26, 2019. In total, 222 patients were screened for eligibility, and 50 patients did not meet the inclusion criteria: no tablet or smartphone (n=17), no proficiency in the Dutch language (n=10), AHI <15 (n=10), history of CPAP treatment (n=5), no OSA (n=4), psychiatric or cognitive disorder (n=3), and age <18 years (n=1). In total, 28 patients declined to participate, and 4 patients were not informed about the study for other reasons: 2 patients were not referred to the researcher due to logistical errors, 1 patient followed a different care process (there was no consultation with the pulmonologist that same day), and 1 patient had had CPAP for try out for a short period.

**Figure 1:**
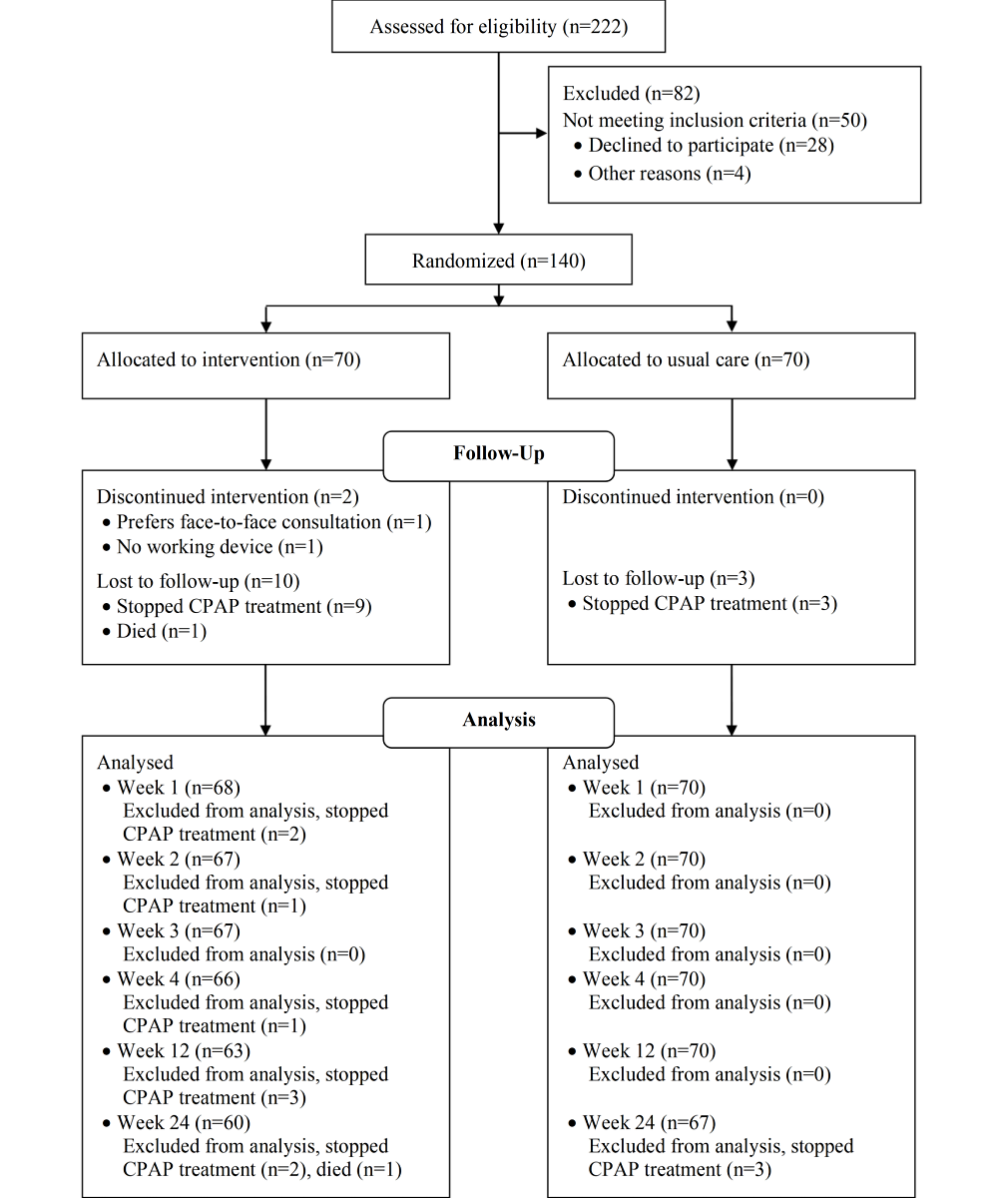
CONSORT flow diagram.

In total, 140 patients were randomized, and 70 patients were allocated to the intervention group and 70 patients to the usual care group. During the intervention period, 2 patients discontinued the intervention: 1 preferred face-to-face consultation, and 1 had no working device. Four patients stopped CPAP treatment during the intervention period (first 4 weeks). In total, 10 patients were lost to follow-up in the intervention group (n=9 stopped CPAP treatment and n=1 died) and 3 in the usual care group (n=3 stopped CPAP treatment). See Figure 1 for the CONSORT (Consolidated Standards of Reporting Trials) flow diagram.

### Baseline Characteristics

Both groups had similar baseline characteristics (Table 1), only outcome expectancies (*P*=.048) and risk perception (*P*=.02) appeared to be significantly different between groups.

**Table 1:** Baseline characteristics (n=140).

Characteristics		All patients (n=140)	Intervention (n=70)	Usual care (n=70)	*P* value
Gender, women, n (%)		29 (21)	12 (17)	17 (24)	.30
Age (years), mean (SD)		53.3 (12.1)	52.3 (12.4)	54.3 (11.9)	.40
AHI^a^, median (IQR)		31.0 (21.5-45.0)	31.0 (22.0-46.0)	30.5 (20.0-42.0)	.96
Living with a partner, n (%)		110 (79)	59 (84)	51 (73)	.10
**Education, n (%)**		—^b^	—	—	.22
	Low	8 (6)	3 (4)	5 (7)	—
	Middle	89 (64)	41 (59)	48 (69)	—
	High	43 (31)	26 (37)	17 (24)	—
**Internet use: duration, n (%)**		—	—	—	>.99
	<6 months	3 (2)	1 (1)	2 (3)	—
	1-2 years	1 (1)	1 (1)	0 (0)	—
	>2 years	1 (1)	1 (1)	0 (0)	—
	>3 years	135 (96)	67 (96)	68 (97)	—
**Internet use: frequency, n (%)**		—	—	—	.31
	(almost) every day	128 (91)	66 (94)	62 (89)	—
	Multiple days per week	9 (6)	4 (6)	5 (7)	—
	≤1 day per week	3 (2)	0 (0)	3 (4)	—
**Tablet or smartphone skills, n (%)**		—	—	—	.91
	Quite bad or bad	5 (4)	2 (3)	3 (4)	—
	Not good or not bad	23 (16)	11 (16)	12 (17)	—
	Quite good	27 (19)	14 (20)	13 (19)	—
	Good	55 (39)	26 (37)	29 (41)	—
	Very good	30 (21)	17 (24)	13 (19)	—
Expects to need help with tablet or smartphone use, n (%)		26 (19)	11 (16)	15 (22)	.41
**Comorbidities, n (%)**		—	—	—	—
	Obesity (BMI >30)	97 (69)	51 (73)	46 (66)	.36
	Hypertension	48 (34)	24 (34)	24 (34)	>.99
	Hypercholesterolemia	21 (15)	8 (11)	13 (19)	.24
	Heart disease	20 (14)	11 (16)	9 (13)	.63
	Diabetes	14 (10)	7 (10)	7 (10)	>.99
**ESS^c^ score, n (%)**		—	—	—	.19
	Total score ≤10	105 (79)	56 (84)	49 (74)	—
	Total score >10	28 (21)	11 (16)	17 (26)	—
**SEMSA^d^ constructs**		—	—	—	—
	Outcome expectancies, mean (SD)	2.78 (0.62)	2.88 (0.57)	2.67 (0.65)	.048
	Self-efficacy, median (IQR)	3.00 (2.56-3.56)	3.00 (2.56-3.33)	3.00 (2.56-3.67)	.40
	Risk perception, median (IQR)	2.00 (1.54-2.50)	2.31 (1.63-2.63)	1.88 (1.50-2.31)	.02

^a^AHI: apnea-hypopnea index.

^b^Not applicable.

^c^ESS: Epworth Sleepiness Scale.

^d^SEMSA: Self-Efficacy Measure for Sleep Apnea.

### CPAP Use

The use of video consultation does not lead to superior results on CPAP use compared with face-to-face consultation. A significant difference in change over time was found between groups for short-term (weeks 1 through 4) CPAP use (*P*-interaction=.008). However, the specific time points (week 1: *P*=.62; week 2: *P*=.15; week 3: *P*=.33, and week 4: *P*=.20) were not significantly different. See Multimedia Appendix 2 and Multimedia Appendix 3 for more detailed information on short-term CPAP use.

**Figure 2:**
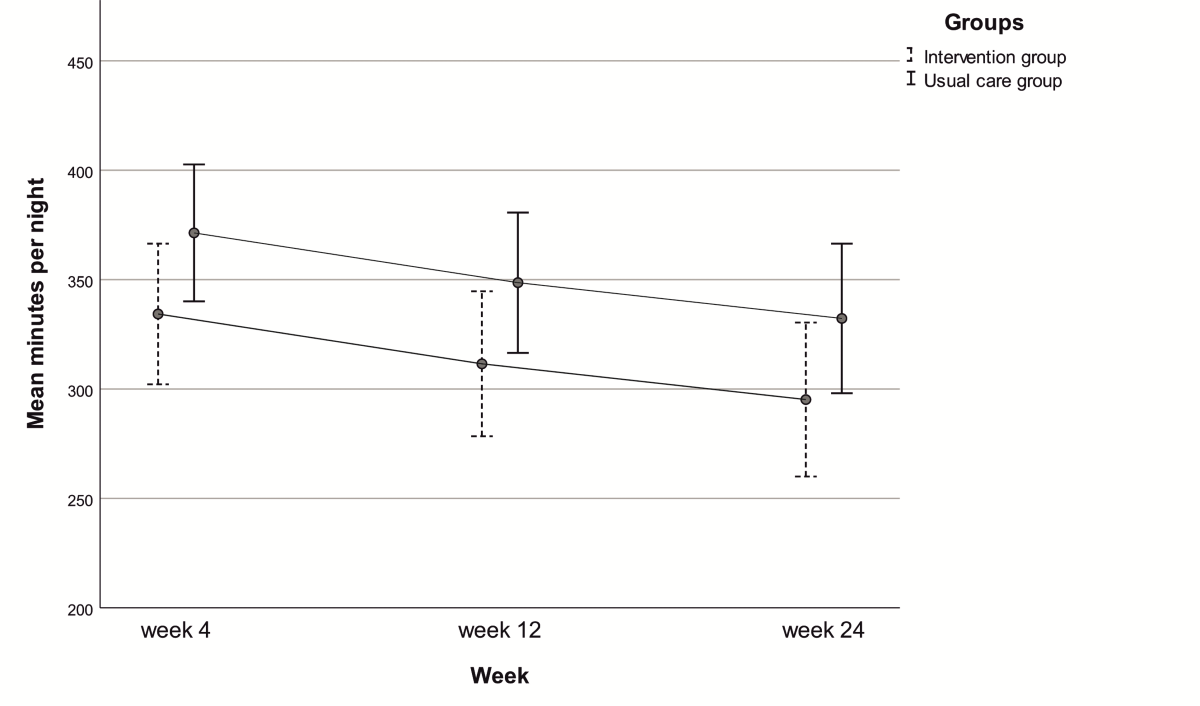
Long-term continuous positive airway pressure use: change over time.

No significant difference in change over time for long-term CPAP use (week 4, week 12, and week 24) was found between groups (*P*-interaction=.68). CPAP use decreased for both groups in the long term (*P*=.008), but no significant difference was found between the intervention and usual care group (*P*=.09). See Table 2 and Figure 2 for change in CPAP use over time (week 4, week 12, and week 24).

**Table 2:** Long-term continuous positive airway pressure use (minutes per night).

Week^a^	Intervention		Usual care	
	EMM^b^ (SE)	95% CI	EMM(SE)	95% CI
Week 4	334.3 (16.3)	302.1-366.5	371.4 (15.8)	340.1-402.7
Week 12	311.5 (16.8)	278.4-344.6	348.6 (16.2)	316.5-380.7
Week 24	295.2 (17.8)	260.0-330.4	332.7 (17.3)	298.1-366.5

^a^Linear mixed model.

^b^EMM: estimated marginal mean.

### CPAP Adherence

The use of video consultation does not lead to superior results on CPAP adherence compared with face-to-face consultation. No significant difference was found between both groups for short-term (*P*=.95) and long-term (*P*=.12) CPAP adherence. Also, no significant difference in change over time between the intervention and usual care group was found for short-term (*P*-interaction=.17) and long-term (*P-*interaction=.51) CPAP adherence. See Multimedia Appendix 4 and Multimedia Appendix 5 for the short-term and long-term adherence rates per week.

### Self-Efficacy, Risk Outcomes, and Outcome Expectancies

No significant difference between groups was found for the SEMSA constructs: outcome expectancies (*P*=.64), self-efficacy (*P*=.41), and risk perception (*P*=.30). See Multimedia Appendix 6.

### Relation Between Self-Efficacy, Risk Perception, Outcome Expectancies, and CPAP Use

After 4 weeks, a relation was found between CPAP use and self-efficacy (*P*=.001), meaning that patients with higher levels of self-efficacy showed higher CPAP use. There was no relation between CPAP use and risk perception (*P*=.34) or outcome expectancies (*P*=.76). Also, the difference between the intervention and usual care group was not significant (*P*=.25).

### Expectations and Experiences With Video Consultation

Patients expressed positive expectations for the use of video consultation. After 4 weeks, 76% (48/63) indicated that video consultation had a positive effect on control over their treatment, and 75% (47/63) indicated that it positively affected the treatment itself. The majority (58/63, 92%) implied it did not cost them effort, 95% (60/63) reported that they had enough skills to use a tablet or smartphone and that they received enough support (53/63, 84%). Although, 64% (44/69) expected to be stimulated by people in their direct environment to use video consultation, only 25% (16/63) were actually stimulated. Almost all patients (60/63, 95%) intended to keep using video consultation. See Multimedia Appendix 7.

### Satisfaction With Consultation

Patients in both groups were satisfied with the consultations. On average, the intervention group rated the consultations with an 8.5 and the usual care group with an 8.3 on a scale of 1 to 10 (1=not at all satisfied to 10=very satisfied). Patients indicated (intervention group versus usual care group) that health care professionals understood their problems (59/63, 94%, vs 58/68, 85%) and listened to them (60/63, 95%, vs 61/68, 90%). Almost all patients understood the content of the consultation (61/63, 97%, vs 62/68, 91%), could easily express their feelings (59/63, 94%, vs 62/68, 91%), and were satisfied with the information they received (58/63, 92%, vs 60/68, 88%). However, more patients with video consultation reported that they did not miss important information (56/63, 89%, vs 43/68, 63%). See Multimedia Appendix 8.

### Satisfaction With Video Consultation

The majority (56/63, 89%) of the patients were very satisfied with video consultation, the quality of the video (50/63, 79%), and sound of the system (45/63, 71%). It also saved them time (61/63, 97%) and provided better access to health care professionals (43/63, 68%). Almost all patients felt safe about their privacy and confidentiality (61/63, 97%) and preferred a video consultation over a face-to-face consultation (51/63, 81%). According to almost half (28/63, 44%) the patients, face-to-face consultation can be replaced by video consultation. See Multimedia Appendix 9.

### Nurse Satisfaction

Nurses (n=3) rated the use of video consultation on average with a 7.3 (SD .57) on a scale of 1 to 10 (1=not at all satisfied to 10=very satisfied). They were all satisfied with privacy and confidentiality and quality of the sound and video and would recommend its use to colleagues and patients. Two nurses agreed that its use fits in their work process. However, only one nurse was completely satisfied with the information she could provide. They did not think that the use of video consultation helped them save time or work more efficiently.

The nurses reported that use of video consultation is not suitable for new patients, and they prefer to use it during follow-up:

It is not suitable for a first consultation after starting CPAP because you cannot provide enough information.

Not for new patients because providing information and checking the device and sleep mask is difficult using video consultation.

The nurses also experienced some technical problems:

Sometimes there were log-in problems and I had to call the patient first by phone.

Sometimes it took long before there was a connection. This costs more time.

They also provided suggestions for improvement and described advantages of video consultations:

Plan the video consultations one after the other and not alternating with face-to-face consultations.

It is a good alternative for follow-up consultations. It is more patient friendly than a face-to-face consultation.

Saves time for patients.

## Discussion

### Principal Findings

In this RCT, we evaluated the superiority of video consultation over face-to-face consultation for newly diagnosed OSA patients. For CPAP use, we found a significant difference in change over time between groups in the short term (*P-*interaction=.008). However, the specific time points (week 1: *P*=.62; week 2: *P*=.15; week 3: *P*=.33, and week 4: *P*=.20) were not significantly different. No significant difference in change over time was found for long-term CPAP use (*P-*interaction=.68). No significant difference in change over time between groups was found for short-term (*P-*interaction=.17) or long-term (*P-*interaction=.51) CPAP adherence. Self-efficacy appeared to have a statistically significant effect on CPAP use in both groups (*P*=.001) regardless of the intervention arm (*P*=.25). No significant difference between groups was found for outcome expectancies (*P*=.64), self-efficacy (*P*=.41), or risk perception (*P*=.30). The experiences with video consultation were very positive. Almost all patients (60/63, 95%) intended to keep using video consultation. Patients in both groups rated the consultations on average with an 8.4. All nurses (n=3) were satisfied with privacy and confidentiality aspects and quality of the sound and video. However, they expressed some recommendations for improvement (eg, to use video consultation only in follow-up).

### Comparison With Prior Work

Unfortunately, change over time was not evaluated in previous controlled studies [18,26,29], but this evaluation is as such a likely pattern. In our study, a significant difference in CPAP use between video consultation and face-to-face consultation was not found. Parikh et al [18] reported statistically equivalent CPAP use for new OSA patients (mean average use minutes per day 305.31 vs 340.55, *P*=.15). In a multicenter RCT, no statistically significant difference was found for CPAP use after 6 months (telemedicine mean use 4.4 [SD 2.0] hours per day vs face-to-face 4.2 [SD 2.0] hours per day, *P*=.83) and adherence (telemedicine 65% vs usual care 57% compliance, *P*=.33) [29]. Based on these findings, it appears that CPAP use is equivalent to using video consultation.

Where previous studies mainly focused on CPAP use, adherence, and satisfaction with video consultation [17,18,25,26,29], we additionally evaluated the combination of cognitive components (self-efficacy, outcome expectancies, and risk perception), experience with the technology (using the UTAUT model), and satisfaction of patients and nurses. This combination of outcomes has received little attention until now. Cognitive components are found to be increasingly important in predicting CPAP use [13,14,27]. Our results show that use of CPAP is higher in patients with high levels of self-efficacy (*P*=.001) regardless of the intervention arm (*P*=.25). In order to improve self-efficacy, it is necessary to positively influence patient perceptions. Patients may benefit from a self-management approach [27,41,42] with tailored education to change their perceptions about CPAP use and subsequently improve self-efficacy [43]. Lai et al [44] provided patients with additional education to enhance, for example, self-efficacy. This increased CPAP use compared with patients receiving usual care (*P*<.001). Stepnowsky et al [41] showed that a self-management program with information about OSA- and CPAP-related issues led to high self-efficacy scores (4.5 [SD 0.6]; scale 0 to 5) and CPAP adherence (5.5 [SD 2.3] mean hours per night). Because self-efficacy scores can be affected by the time that patients are treated, scores should be assessed regularly in order to be useful in clinical practice [14].

However, limited evidence was available about the effect of video consultation for newly diagnosed patients starting CPAP. Most previous RCTs were small, with sample sizes varying from 19 to 40 patients [17,25]. Only Isetta et al [29] evaluated CPAP compliance with a fully powered sample size. Although almost half of the patients (40%) in this study had insufficient digital skills, technology aspects were not evaluated [29]. In our study, 9% (20/222) were unable to participate because of lack of access to a mobile device or due to psychiatric or cognitive disorder. During the intervention, 2 patients (2/70, 3%) discontinued the video consultation intervention because of preference for face-to-face consultation or problems with their mobile device. The use of video consultation is evolving rapidly in clinical practice, but digital services are not applicable to all patients and digital health literacy remains a challenge [45]. This is especially due to lack of awareness or knowledge or unwillingness to change [46] and emphasizes the importance of personalized interventions rather than a one-size-fits-all approach.

The assessment of UTAUT components and self-efficacy can also be used to indicate technology use [47]. To our knowledge, no previous studies have identified technology acceptance for OSA patients using video consultation. Patients in our study had positive experiences with the use of video consultation and were satisfied with the video consultation system and consultations in general. Previous studies also reported high satisfaction scores [17,18,25,26], mostly regarding communication with a health care professional [18] and privacy and security factors [17]. Although most patients would recommend the use of video consultations to others, not all patients in our study are convinced that all visits can be replaced by video consultations. This is in line with findings from previous research [17].

The involvement of health care professionals is essential to achieve successful implementation of technology [48], but this is often not evaluated [17]. We found that nurses (n=3) preferred to start with a face-to-face consultation because education about the sleep mask and adjustments are often required during the first follow-up appointment with the nurse. The applicability of technology use may be dependent on the population [49], and for OSA patients, the use of video consultation in a blended care setting might therefore be beneficial. We found that the nurses were satisfied with video consultation and especially with the quality of the system, privacy and confidentiality. They would recommend it to colleagues and patients. Nurses also reported technical problems (eg, problems with Wi-Fi connections). Technological issues are often seen as a barrier [50], and it is important to take technical elements into account [48,51,52] during implementation. Another point for improvement is integration in existing health care processes (eg, planning). To achieve successful implementation, it can be beneficial to involve professionals during the implementation process itself [50].

Video consultation can be seen as a promising app to support OSA patients during treatment. Still, evidence was lacking and previous research was not strong enough in design or focused on a limited number of outcomes. With the evaluation of a broad range of outcomes affecting CPAP use and implementation of video consultation in clinical practice, this RCT adds value to current knowledge.

However, proper evaluation in this field is challenging because research often lags behind the rapid development of technology [53]. The use of pragmatic trials may be promising [54] to evaluate different elements of eHealth solutions in a hospital setting and can, for example, be used to get (more) rapid insights in relevant implementation outcomes such as feasibility, impact on an organization, and acceptance and adoption by health care professionals and patients. Future research should focus on blended care approaches in which self-efficacy especially receives greater emphasis. For organizations to be able to implement video consultation on a larger scale, integration in existing health care processes and technology acceptance by patients and professionals is necessary.

### Limitations

Several limitations should be considered. Risk perception and outcome expectancies were significantly different at baseline, despite randomization. For a limited number of patients (7/66, 11%, in the intervention group and 6/70, 9%, in the control group), video consultations or face-to-face consultations were replaced with a telephonic consultation due to technical problems in the intervention group and because patients in the control group could not come to the hospital. The protocol process were not strictly followed because patients failed to attend their scheduled appointment (no show, sick, on holiday) or there were organizational inaccuracies such as wrongly scheduled appointments. The percentage of patients that followed the process exactly as described (Multimedia Appendix 1) was higher in the intervention group (approximately half) than in the usual care group (approximately one-third). However, all patients received the intervention (type of consultation) they were allocated to except for the 2 patients who discontinued the intervention (Figure 1). Another limitation is that only 3 nurses were involved in the evaluation. Therefore, a firm conclusion on professional aspects cannot be drawn.

### Conclusion

Support of OSA patients with video consultation does not lead to superior results on CPAP use and adherence compared with face-to-face consultation. The findings of this research show that a significant difference in change over time was found between groups for short-term CPAP use (but not on specific time points), but not for long-term CPAP use. Levels of self-efficacy were positively related to CPAP use in both groups. Patients were very satisfied with video consultation and reported positive experiences.

Therefore, the findings of this research suggest that self-efficacy is an important factor in improving CPAP use and that video consultation may be a feasible way to support patients starting CPAP. The integration in health care processes and tailoring video consultation use to patient and professional needs is essential to ensure successful use. A blended care setting, in which an initial video consultation is combined with face-to-face consults, may be beneficial. To our knowledge, this is the first RCT that examined the effects of video consultation on CPAP use over time for newly diagnosed OSA patients in combination with cognitive components and experience with technology use. Future research should focus on blended care approaches in which self-efficacy receives greater emphasis.

## Appendix

Multimedia Appendix 1 Study process.

Multimedia Appendix 2 Short-term CPAP use.

Multimedia Appendix 3 Short-term CPAP use: change over time.

Multimedia Appendix 4 Short-term CPAP adherence.

Multimedia Appendix 5 Long-term CPAP adherence.

Multimedia Appendix 6 Self-Efficacy Measure for Sleep Apnea constructs: self-efficacy, risk perception, and outcome expectancies.

Multimedia Appendix 7 Expectations and experiences with video consultation.

Multimedia Appendix 8 Patient satisfaction with consultation.

Multimedia Appendix 9 Patient satisfaction with video consultation.

Multimedia Appendix 10 CONSORT-eHEALTH checklist (V 1.6.1).

## Abbreviations

AHI: apnea-hypopnea index
CONSORT: Consolidated Standards of Reporting Trials
CPAP: continuous positive airway pressure
OSA: obstructive sleep apnea
RCT: randomized controlled trial
SEMSA: Self-Efficacy Measure for Sleep Apnea
UTAUT: unified theory of acceptance and use of technology
