# Supplementary figures and images for: Video Consultation as an Adequate Alternative to Face-to-Face Consultation in Continuous Positive Airway Pressure Use for Newly Diagnosed Patients With Obstructive Sleep Apnea: Randomized Controlled Trial
Source: JMIR Form Res. 2021 May 11;5(5):e20779. doi: 10.2196/20779 (PMC8150406; doi:10.2196/20779)

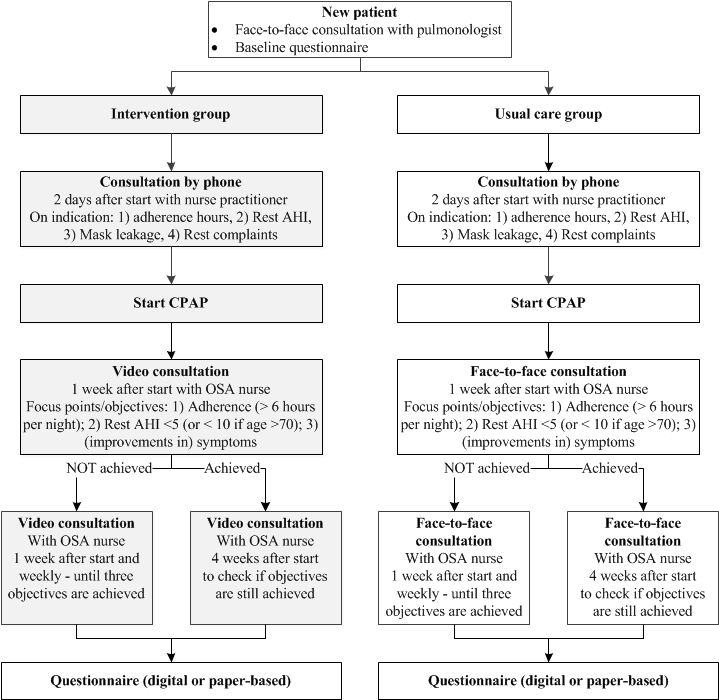

Supplement: Multimedia Appendix 1 [file formative_v5i5e20779_app1.png]

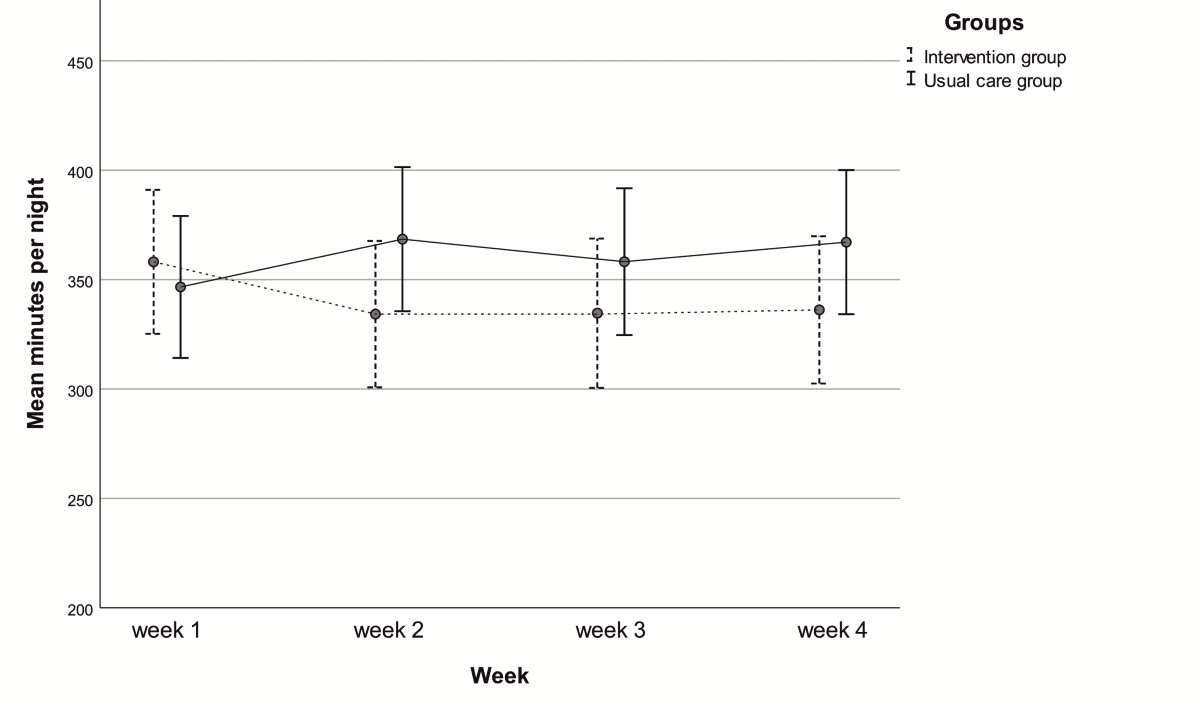

Supplement: Multimedia Appendix 3 [file formative_v5i5e20779_app3.png]
