# Supplementary material for: Video Consultation as an Adequate Alternative to Face-to-Face Consultation in Continuous Positive Airway Pressure Use for Newly Diagnosed Patients With Obstructive Sleep Apnea: Randomized Controlled Trial
Source: JMIR Form Res. 2021 May 11;5(5):e20779. doi: 10.2196/20779 (PMC8150406; doi:10.2196/20779)
Supplement: Multimedia Appendix 2 [file formative_v5i5e20779_app2.doc]

Table 3. Short-term CPAP use (minutes per night)

| Week^a^ | Intervention | | Usual care | |
| --- | --- | --- | --- | --- |
|  | EMM (SE) | 95% CI | EMM (SE) | 95%CI |
| Week 1 | 358.2 (16.7) | 325.2 – 391.1 | 346.7 (16.4) | 314.2 – 379.1 |
| Week 2 | 334.2 (16.9) | 300.8 – 367.7 | 368.5 (16.6) | 335.6 – 401.4 |
| Week 3 | 334.7 (17.3) | 300.6 – 368.8 | 358.2 (17.0) | 324.7 – 391.7 |
| Week 4 | 336.2 (17.0) | 302.6 – 369.8 | 367.2 (16.7) | 334.2 – 400.1 |

^a^ Linear mixed model
