# Supplementary material for: Video Consultation as an Adequate Alternative to Face-to-Face Consultation in Continuous Positive Airway Pressure Use for Newly Diagnosed Patients With Obstructive Sleep Apnea: Randomized Controlled Trial
Source: JMIR Form Res. 2021 May 11;5(5):e20779. doi: 10.2196/20779 (PMC8150406; doi:10.2196/20779)
Supplement: Multimedia Appendix 5 [file formative_v5i5e20779_app5.doc]

Table 5. Long-term CPAP adherence (CPAP use 5 ≥ nights ≥ 4 hours per night)

| Week^a^ | Intervention (%) | Usual care (%) |
| --- | --- | --- |
| Week 4 | 78% | 85% |
| Week 12 | 65% | 75% |
| Week 24 | 63% | 73% |

^a^ Generalized estimating equations
