# Supplementary material for: Video Consultation as an Adequate Alternative to Face-to-Face Consultation in Continuous Positive Airway Pressure Use for Newly Diagnosed Patients With Obstructive Sleep Apnea: Randomized Controlled Trial
Source: JMIR Form Res. 2021 May 11;5(5):e20779. doi: 10.2196/20779 (PMC8150406; doi:10.2196/20779)
Supplement: Multimedia Appendix 6 [file formative_v5i5e20779_app6.doc]

Table 6. Self-efficacy Measure for Sleep Apnea (SEMSA) constructs, after 4 weeks

| SEMSA constructs | Intervention | Usual care | *P* value |
| --- | --- | --- | --- |
| Outcome expectancies, mean (SD)^a^ | 2.89 (.56) | 2.84 (.65) | .64 |
| Self-efficacy, median (IQR)^b^ | 3.00 (2.89 – 3.44) | 3.22 (2.71 – 3.67) | .41 |
| Risk perception, median (IQR)^b^ | 1.75 (1.50 – 2.31) | 1.63 (1.31 – 2.44) | .30 |

^a^ Independent samples t-test
^b^ Mann-Whitney U test
