# Supplementary material for: Video Consultation as an Adequate Alternative to Face-to-Face Consultation in Continuous Positive Airway Pressure Use for Newly Diagnosed Patients With Obstructive Sleep Apnea: Randomized Controlled Trial
Source: JMIR Form Res. 2021 May 11;5(5):e20779. doi: 10.2196/20779 (PMC8150406; doi:10.2196/20779)
Supplement: Multimedia Appendix 7 [file formative_v5i5e20779_app7.doc]

Table 7. Expectations and experiences with video consulation (intervention group)

| Statements based on Unified Theory of Acceptance of Technology | Expectation: baseline (N=70)^a^, n (%) | Experience: after 4 weeks (N=66)^a,b^, n (%) |
| --- | --- | --- |
| I will have more control over my treatment using video consultation | 46 (66) |  |
| I have more control over my treatment using video consultation |  | 48 (76) |
| The use of video consultation will have a positive effect on my treatment | 48 (69) |  |
| The use of video consultation had a positive effect on my treatment |  | 47 (75) |
| It will not cost me effort to use video consultation | 57 (81) |  |
| It did not cost me effort to use video consultation |  | 58 (92) |
| People in my direct environment will stimulate me to use video consultation | 44 (64) |  |
| People in my direct environment stimulated me to use video consultation |  | 16 (25) |
| I have (tablet/smartphone) skills to use video consultation | 64 (91) |  |
| I had (tablet/smartphone) skills to use video consultation |  | 60 (95) |
| I will receive enough support to use video consultation | 60 (86) |  |
| I received enough support to use video consultation |  | 53 (84) |
| I intend to use video consultation | 66 (94) |  |
| I will keep using video consultation |  | 60 (95) |

^a^Number and valid percentage of patients that agree or totally agree (≥5 on 7-point scale, 1: totally disagree to 7: totally agree)

^b^ n=4 patients lost to follow-up and n=3 patients did not complete the questionnaire
