# Supplementary material for: Video Consultation as an Adequate Alternative to Face-to-Face Consultation in Continuous Positive Airway Pressure Use for Newly Diagnosed Patients With Obstructive Sleep Apnea: Randomized Controlled Trial
Source: JMIR Form Res. 2021 May 11;5(5):e20779. doi: 10.2196/20779 (PMC8150406; doi:10.2196/20779)
Supplement: Multimedia Appendix 8 [file formative_v5i5e20779_app8.doc]

Table 8. Patient satisfaction with consultation, after 4 weeks

| Satisfaction statements | Intervention (N=66)^a,b^, n(%) | Usual care (N=70)^a,c^,  n(%) |
| --- | --- | --- |
| The health care professional understood my problems | 59 (94) | 58 (85) |
| The health care professional listened to me during the (video/face-to-face) consultations | 60 (95) | 61 (90) |
| It was easy to express my feelings during the (video/face-to-face) consultations | 59 (94) | 62 (91) |
| I am satisfied with the information that I received during the (video/face-to-face) consultations | 58 (92) | 60 (88) |
| I did not miss important information during the (video/face-to-face) consultations | 56 (89) | 43 (63) |
| The explanation that I received during the (video/face-to-face) consultations helped me | 55 (87) | 58 (85) |
| I understood the content of the (video/face-to-face) consultations | 61 (97) | 62 (91) |
| I felt comfortable during the (video/face-to-face) consultations | 60 (95) | 62 (91) |

^a^ Number and valid percentage of patients that agree or totally agree (≥5 on 7-point scale, 1: totally disagree to 7: totally agree)

^b^ n=4 patients lost to follow-up and n=3 patients did not complete the questionnaire

^c^ n=2 patients did not complete the questionnaire
