# Supplementary material for: Video Consultation as an Adequate Alternative to Face-to-Face Consultation in Continuous Positive Airway Pressure Use for Newly Diagnosed Patients With Obstructive Sleep Apnea: Randomized Controlled Trial
Source: JMIR Form Res. 2021 May 11;5(5):e20779. doi: 10.2196/20779 (PMC8150406; doi:10.2196/20779)
Supplement: Multimedia Appendix 9 [file formative_v5i5e20779_app9.doc]

Table 9. Patient satisfaction with video consultation, after 4 weeks

| Satisfaction with video consultation statements | Intervention (N=66)^a^, n(%) |
| --- | --- |
| I save time because of video consultation | 61 (97)^b^ |
| I felt safe about my privacy and confidentiality | 61 (97)^b^ |
| I would recommend video consultation to patients in a similar situation | 57 (91)^b^ |
| I am (very) satisfied with the use of video consultation | 56 (89)^b^ |
| I prefer a consult with video consultation than face-to-face) | 51 (81)^b^ |
| I am satisfied with the quality of the video | 50 (79)^b^ |
| I am satisfied with the quality of the sound | 45 (71)^b^ |
| I have better access to my health care professionals because of video consultation | 43 (68)^b^ |
| I think that a video consultation can replace all consultations in the hospital | 28 (44)^c^ |

^a^ n=4 patients lost to follow-up and n=3 patients did not complete the questionnaire

^b^ Number and valid percentage of patients that agree or totally agree (≥5 on 7-point scale, 1: totally disagree to 7: totally agree)

^c^ Yes/No/Maybe question: percentage of patients that answered ‘yes’
